# Glymphatic, Structural, and Cognitive Changes During Breast Cancer Chemotherapy: A Longitudinal MRI Study

**DOI:** 10.1002/hbm.70334

**Published:** 2025-08-29

**Authors:** Xiaoyu Zhou, Yixin Hu, Jing Yang, Yao Huang, Hua Lan, Jiahui Zheng, Lin Tang, Jing Zhang, Jun Chen, Ting Yin, Daihong Liu, Jiuquan Zhang

**Affiliations:** ^1^ School of Medicine Chongqing University Chongqing China; ^2^ Chongqing Key Laboratory for Intelligent Oncology in Breast Cancer, Department of Radiology Chongqing University Cancer Hospital Chongqing China; ^3^ Bayer Healthcare Wuhan China; ^4^ MR Research Collaborations Siemens Healthineers Ltd. Chengdu China

**Keywords:** chemotherapy‐related cognitive impairment, choroid plexus, glymphatic system, MRI, perivascular space

## Abstract

The glymphatic system maintains brain homeostasis through cerebrospinal fluid transport and waste clearance. Its potential involvement in chemotherapy‐related cognitive impairment remains largely unexplored due to limited in vivo evidence. In this prospective longitudinal study, 126 female breast cancer patients underwent multiparametric brain MRI and neuropsychological assessments at three time points: baseline (bc1), after the first cycle of neoadjuvant chemotherapy (bc2), and upon completion of neoadjuvant chemotherapy (bc3). Glymphatic function was assessed using four MRI‐derived metrics: choroid plexus (CP) volume, perivascular space (PVS) volume fraction, free water (FW), and Diffusion Tensor Imaging–Along the Perivascular Space (DTI‐ALPS) index. Brain tissue segmentation was conducted to quantify the volume fractions of gray matter (GM) in cortex and subcortex, white matter (WM), and cerebrospinal fluid (CSF) relative to intracranial volume. Neuropsychological assessments included the Self‐Rating Anxiety Scale (SAS), the Functional Assessment of Cancer Therapy–Cognitive Function (FACT‐Cog), and a battery of objective cognitive tests. Longitudinal changes and interrelationships were analyzed using linear mixed‐effects models, correlation analyses, and cross‐lagged panel analysis. During chemotherapy, CP volume increased (*p* < 0.001), while PVS volume fraction decreased (*p* = 0.003); no significant changes were found in FW or DTI‐ALPS. GM volumes in both cortex and subcortex declined (both *p* = 0.02). SAS scores increased (*p* = 0.02), and FACT‐Cog scores decreased (*p* < 0.001), with no significant changes in objective test scores. From bc2 to bc3, increases in CP volume were negatively correlated with reductions in PVS volume fraction (*r* = −0.40, *p* < 0.001). From bc1 to bc3, reductions in PVS volume fraction were associated with decreases in both cortical GM volumes (*r* = 0.32, *p* < 0.001). At bc2, cortical GM atrophy was correlated with increased SAS scores (*r* = −0.30, *p* = 0.002). Cross‐lagged panel analysis showed that CP enlargement at bc2 preceded PVS volume fraction reduction at bc3 (*β* = −1.66, *p* = 0.007). During neoadjuvant chemotherapy, breast cancer patients exhibited a unique pattern of glymphatic system alterations, suggesting its potential as an imaging marker of treatment‐related brain changes.

## Graphical abstract

Using longitudinal multiparametric glymphatic MRI metrics, we found early choroid plexus enlargement and subsequent perivascular space narrowing in breast cancer patients undergoing neoadjuvant chemotherapy. These changes were associated with brain atrophy, suggesting a sequential disruption of glymphatic pathways that may contribute to chemotherapy‐related brain changes.

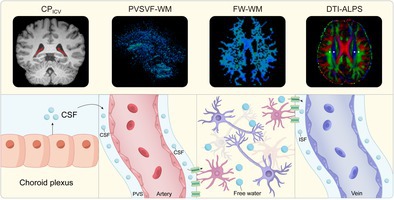

**Abbreviations**
BBB: blood‐brain barrier
bc1: baseline (pre‐treatment)
bc2: after the first cycle of neoadjuvant chemotherapy
bc3: after completion of neoadjuvant chemotherapy but prior to surgery
BCSFB: blood‐cerebrospinal fluid barrier
BMI: body mass index
CP_ICV_
: choroid plexus volume as a ratio relative to the total intracranial volume
CSF: cerebrospinal fluid
CSF/ICV: ratio of cerebrospinal fluid volume to total intracranial volume
DST: Digit Span Test
DTI‐ALPS: Diffusion Tensor Imaging–Along the Perivascular Space
FACT‐Cog: Functional Assessment of Cancer Therapy‐Cognitive Function
FLAIR: fluid‐attenuated inversion recovery
FW: free water
FW‐WM: mean FW in white matter excluding PVS regions
GM_cortex/ICV: ratio of cortical gray matter volume to total intracranial volume
GM_subcortex/ICV: ratio of subcortical gray matter volume to total intracranial volume
ICV: total intracranial volume
ISF: interstitial fluid
LGA: Lesion Growth Algorithm
LMEs: linear mixed‐effects models
MAP: mean arterial pressure
MPRAGE: three‐dimensional magnetization prepared rapid gradient echo
MRS: magnetic resonance spectroscopy
PVS: perivascular space
PVSVF‐WM: white matter PVS volume fraction
SAS: Self‐Rating Anxiety Scale
SDS: Self‐Rating Depression Scale
SPACE: sampling perfection with application optimized contrasts
TMT‐A: Trail Making Test Part A
VFT: Verbal Fluency Test
WM/ICV: ratio of white matter volume to total intracranial volume
WMH: white matter hyperintensity

## 1 Introduction

With the 5‐year survival rate of breast cancer now reaching 91%, increasing attention has been directed toward treatment‐related complications (Siegel et al. 2023). One major concern is chemotherapy‐related cognitive impairment, often referred to as “chemo‐brain,” which affects approximately 17%–75% of patients (Lange et al. 2019). Understanding how chemotherapeutic agents impact the brain has therefore become a critical area of research.

Chemotherapeutic agents widely used in breast cancer, such as anthracyclines and taxanes, exhibit limited permeability across the blood–brain barrier (BBB) or the blood–cerebrospinal fluid barrier (BCSFB) due to their high molecular weights and low lipid solubility (Eide and Feng 2020; Solár et al. 2020). A growing body of evidence suggests that these drugs may affect intracranial homeostasis either by directly damaging these barriers or indirectly through the release of tumor‐derived inflammatory mediators that cross these barriers and alter the brain microenvironment (Fleming et al. 2023).

Within this context, the glymphatic system has recently gained attention as a critical regulator of brain homeostasis (Hsu et al. 2023; Hsiao et al. 2023). It mediates the clearance of metabolic waste via a series of processes: cerebrospinal fluid (CSF), primarily produced by the choroid plexus, enters the perivascular space (PVS) along arteries, exchanges with interstitial fluid (ISF), and drains along venous perivascular pathways (Shen 2018). The choroid plexus, as a central component of the BCSFB, not only produces CSF but also contributes to immune regulation by mediating immune cell and molecular exchange between the periphery and the brain (Xu, Lotfy, et al. 2024). Emerging evidence suggests that the choroid plexus could be sensitive to chemotherapy, although direct evidence remains limited (Jang et al. 2022). Similarly, the PVS, which is anatomically and functionally linked to the BBB, may also have the potential to be affected by chemotherapy‐related changes (Lyu et al. 2021). These considerations highlight the need to further investigate whether and how the glymphatic system may be involved in chemotherapy‐related brain alterations.

Despite this hypothesis, whether the glymphatic system is affected by chemotherapy remains poorly understood. Prior studies have primarily focused on structural or functional brain alterations (Zhou et al. 2022; Yang et al. 2025; de Ruiter et al. 2021; Yao et al. 2023), while only limited work has directly assessed glymphatic function (Gao et al. 2024). Notably, previous research utilizing a single imaging biomarker—Diffusion Tensor Imaging—Along the Perivascular Space (DTI‐ALPS) index—did not reveal significant changes post‐chemotherapy (Gao et al. 2024). Although DTI‐ALPS has been proposed as a biomarker for glymphatic function, it primarily reflects directional water diffusivity along medullary veins and may also be influenced by broader perivascular and microstructural properties (Taoka et al. 2017). This highlights the need for a more comprehensive, multiparametric MRI approach (Taoka et al. 2024).

**FIGURE 1:**
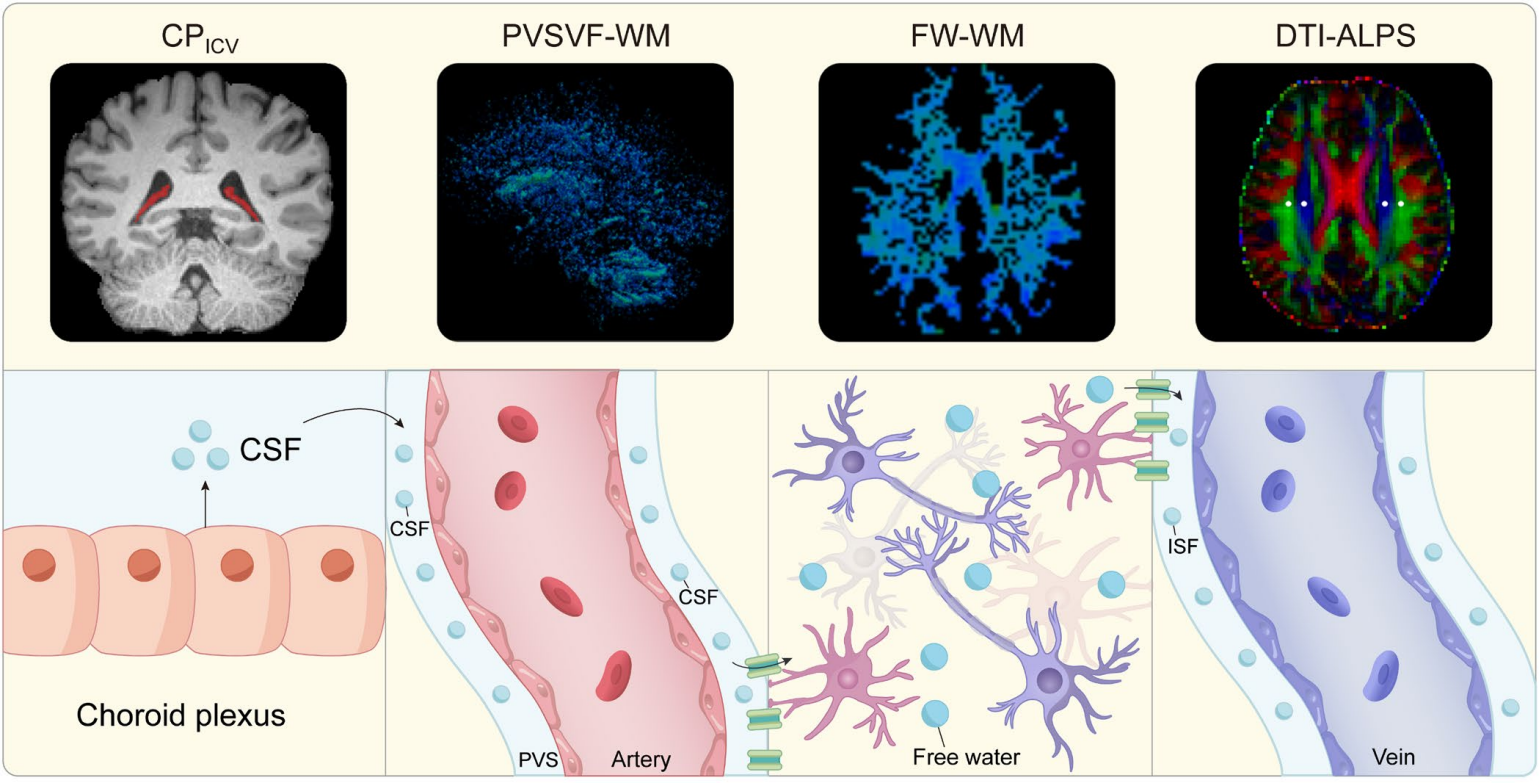
Schematic illustration of the four physiological processes of the glymphatic system and their corresponding MRI‐derived metrics. (i) choroid plexus producing cerebrospinal fluid (indexed by CP_TIV_, ratio of choroid plexus volume to intracranial volume), (ii) cerebrospinal fluid (CSF) influx into perivascular spaces (indexed by PVSVF‐WM, volume fraction of perivascular space in white matter), (iii) exchange of CSF and interstitial fluid (ISF) (indexed by FW‐WM, volume fraction of free water in white matter), and (iv) waste clearance (indexed by DTI‐ALPS, diffusivity along the perivascular space).

Recent advances in imaging now allow for non‐invasive assessment of the glymphatic system's four key steps: CSF production, perivascular inflow, interstitial exchange, and clearance (Figure 1). Disruption of these processes has been implicated in neurodegenerative and vascular diseases such as Alzheimer's disease (Hsu et al. 2023; Kamagata et al. 2022; Choi et al. 2022; Ray et al. 2023), Parkinson's disease (Scott‐Massey et al. 2022), multiple sclerosis (Carotenuto et al. 2022), and cerebral small vessel disease (Xu et al. 2022).

Building on this framework, we conducted a prospective longitudinal study to assess glymphatic function in breast cancer patients undergoing neoadjuvant chemotherapy. Using a multiparametric MRI strategy, we systematically evaluated the four major components of glymphatic flow and examined their associations with brain structural changes and cognitive outcomes. Importantly, we also sought to explore the potential temporal sequence of these changes—for example, whether alterations in the choroid plexus precede changes in perivascular spaces. These findings may offer new insights into the pathophysiology of chemo‐brain and help identify novel targets for early intervention and future research.

## 2 Materials and Methods

### 2.1 Study Participants

This prospective study protocol was approved by the institutional review board, and all participants provided written informed consent prior to enrollment.

Between February 2021 and September 2023, we consecutively enrolled female breast cancer patients at three time points: baseline (pre‐treatment, bc1), after the first cycle of neoadjuvant chemotherapy (bc2), and after completion of neoadjuvant chemotherapy but prior to surgery (bc3). All participants underwent multiparametric brain MRI and cognitive evaluations at a minimum of two timepoints, which allowed for valid longitudinal analysis using mixed‐effects models.

The inclusion criteria were as follows: (i) female patients with histopathologically confirmed breast cancer; and (ii) those with stage II or III cancer who are scheduled to receive or have undergone neoadjuvant chemotherapy before surgery. The exclusion criteria were as follows: (i) brain structural abnormalities (e.g., metastases) identified based on T1‐weighted and T2 FLAIR MRI sequences and diagnosed by experienced neuroradiologists; (ii) severe white matter hyperintensity, defined as a Fazekas score of 3; (iii) psychiatric disorders or being left‐handed; (iv) serious chronic medical conditions (e.g., chronic kidney disease, autoimmune disorders); (v)changing treatment strategies or refusing subsequent MRI scans twice; and (vi) poor quality imaging.

### 2.2 Neuropsychological Evaluation

All participants completed a standardized neuropsychological battery (Zhou et al. 2022) including subjective and objective aspects. Subjective evaluation includes the Self‐Rating Anxiety Scale (SAS), the Self‐Rating Depression Scale (SDS), and the Functional Assessment of Cancer Therapy‐Cognitive Function (FACT‐Cog) (Tiksnadi et al. 2023; Von Ah and Tallman 2015). Objective evaluation includes the Digit Span Test (DST, forward and backward), the Trail Making Test Part A (TMT‐A), and the Verbal Fluency Test (VFT) for assessing memory, attention, executive function, and mental flexibility (Zhang et al. 2020; Onyedibe et al. 2025; Andryszak et al. 2017).

### 2.3 MRI Acquisition

This study used a 3.0 T MRI scanner (Magnetom Prisma, Siemens Healthcare) with a 64‐channel head coil. Participants wore earplugs to reduce scanner noise, and foam cushions were placed around the head within the coil to minimize motion artifacts. All scans were performed during the daytime, and participants were instructed to remain awake throughout the scanning session (Han et al. 2023). The MRI protocol included the following sequences: (i) sagittal 3D T1‐weighted volume scans were obtained using the three‐dimensional magnetization prepared rapid gradient echo (MPRAGE) sequence, inversion time [TI] = 900 milliseconds, repetition time [TR] = 2100 milliseconds, echo time [TE] = 2.26 milliseconds, flip angle = 8°, field of view [FOV] = 256 × 256 mm^2^, matrix = 256 × 256, slice thickness = 1 mm with no slice gap, voxel size = 1 × 1 × 1 mm^3^ and slices = 192; (ii) axial pulsed‐gradient spin echo single shot diffusion‐weighted echo planar imaging was performed along 30 gradient directions for b = 1000 s/mm^2^ and another 30 gradient directions for b = 2000 s/mm^2^, with an additional acquisition of 10 volumes at b = 0 s/mm^2^, TE = 76 milliseconds, TR = 3400 milliseconds, voxel size = 2.0 × 2.0 × 2.0 mm^3^, FOV = 220 × 220 mm^2^ and slices = 60; (iii) 3D T2‐weighted fluid‐attenuated inversion recovery (FLAIR) images were acquired using sagittal sampling perfection with application optimized contrasts (SPACE) sequence with the following parameters: TR = 5000 milliseconds, TE = 394 milliseconds, TI = 1600 milliseconds, turbo factor = 192, FOV = 242 × 242 mm^2^, matrix size = 256 × 256, voxel size = 0.5 × 0.5 × 1.0 mm^3^, slices = 160, and 2 averages.

### 2.4 MRI Analysis

#### 2.4.1 Blinded Analysis

When analyzing the images, the radiologists were blinded to the participants' age, time points, cognitive assessment outcomes, and clinical information. Image pre‐processing, CP segmentation, PVS segmentation, and free water (FW) and DTI‐ALPS index calculation were performed by one radiologist (X. Zhou, with 5 years of experience in neuroimaging). These generated maps were quality‐checked independently by a second radiologist (D. Liu, with 12 years of experience in neuroimaging).

#### 2.4.2 Image Pre‐Processing

T1‐weighted images were processed using the longitudinal pipeline of the Computational Anatomy Toolbox (CAT12, http://www.neuro.uni‐jena.de/cat/) implemented in SPM12. All T1 images were bias‐corrected, skull‐stripped, and segmented into gray matter, white matter, and cerebrospinal fluid. To improve longitudinal sensitivity, intra‐subject registration was performed using DARTEL‐based diffeomorphic normalization, generating a midpoint image for each participant and aligning all timepoints to this common template. To enhance anatomical specificity, gray matter was further parcellated into cortical and subcortical regions. In addition, intracranial volume (ICV) and lateral ventricular volumes were extracted from the segmented images and used for normalization or covariate adjustment in subsequent analyses.

White matter hyperintensities (WMHs) were segmented using the Lesion Growth Algorithm (LGA, https://www.applied‐statistics.de/lst.html) implemented in the LST toolbox of SPM12. In our analysis, both high‐resolution T1‐weighted and T2‐FLAIR images were provided as inputs to the algorithm. The resulting WMH masks were used to exclude lesion‐overlapping voxels from subsequent analyses.

Diffusion‐weighted imaging data were preprocessed in FSL 6.0.7 (https://fsl.fmrib.ox.ac.uk/fsl/docs/#/) with eddy current correction, brain extraction, and spatial normalization to T1 space.

#### 2.4.3 Choroid Plexus Volumetry

Choroid plexus volume indirectly reflects the function of the choroid plexus in producing cerebrospinal fluid. A deep learning framework nnU‐Net (Isensee et al. 2021) was trained on 30 manually segmented T1 images that were randomly selected, with remaining scans processed via the automated pipeline followed by expert visual correction. To reduce inter‐participant variability, the choroid plexus volume was presented as a ratio relative to the total intracranial volume (CP_ICV_) (Choi et al. 2022).

#### 2.4.4 PVS Quantification

The white matter PVS volume fraction (PVSVF‐WM) indirectly reflects the flow of cerebrospinal fluid into the perivascular spaces. To compute this measure, PVS maps were generated based on the observation that perivascular regions exhibit higher intensities within white matter voxels. The Frangi filter was implemented using the Scikit‐Image library (https://scikit‐image.org/, version 0.19.1), with default parameters (α = 0.5, β = 0.5, C = half the maximum Hessian norm) consistent with previous studies (Kamagata et al. 2022; Lin et al. 2024). Vesselness was computed across multiple scales (0.1–5 voxels) and used to generate the PVS map through maximum likelihood estimation. To reduce the influence of outliers, a standard normalization and binarization procedure was applied, resulting in a binary PVS mask. The white matter mask, with WMH excluded, was used to constrain the PVS mask, ensuring analysis was restricted to normal‐appearing white matter. PVSVF‐WM was finally calculated as the volume of PVS within the corrected white matter mask, normalized to the total intracranial volume.

#### 2.4.5 FW Mapping

The white matter free water volume fraction (FW‐WM), which reflects extracellular fluid accumulation and exchange processes, was derived using Dipy's two‐compartment model (https://dipy.org/) based on preprocessed diffusion‐weighted images (Hoy et al. 2014). Masks for FW calculation were obtained by subtracting the PVS masks from the white matter masks (excluding WMHs) and then transformed into individual DWI space. The resulting FW maps were constrained to these masks, and the total FW‐WM was calculated and normalized to the intracranial volume.

#### 2.4.6 DTI‐ALPS Index

The DTI‐ALPS index reflects glymphatic clearance by measuring water diffusivity along perivascular spaces in white matter near the lateral ventricles. Following established protocols (Taoka et al. 2017; Shen et al. 2022), diffusion‐weighted images with b‐values of 0 and 1000 s/mm^2^ were selected for DTI‐ALPS index analysis using the publicly available alps.sh script (https://github.com/gbarisano/alps). This approach automatically places ROIs based on standardized anatomical landmarks and diffusion directions, enhancing reproducibility across participants and timepoints (Liu et al. 2024). The ROI diameter was set to 4 mm, given the voxel size of 2 × 2 × 2 mm^3^ in the acquired sequences. To avoid lesion‐related confounding, WMH masks were overlaid on the DTI images, and all automatically placed ROIs were visually confirmed to be free from overlap with white matter hyperintensities.

#### 2.4.7 Structural Brain Volumetry

The ICV, computed during image preprocessing, was used as scaling factors for volumetric normalization. To quantify relative tissue distribution, we calculated normalized compartment ratios, including WM/ICV (ratio of white matter volume to ICV), GM_cortex/ICV (ratio of cortical gray matter volume to ICV), GM_subcortex/ICV (ratio of subcortical gray matter volume to ICV), and CSF/ICV (ratio of cerebrospinal fluid volume to ICV). These ratios represent the proportional volumetric contributions of each neuroanatomical compartment to total intracranial space.

### 2.5 Statistical Analysis

All analyses were performed using R 4.4.2, with a two‐tailed significance threshold set at *p* < 0.05. To evaluate the representativeness of the included cohort and assess potential selection bias, ANOVA or the Kruskal–Wallis H tests were used to compare demographic and clinical characteristics between the total cohort and patients at each time point. Similarly, two‐sample *t*‐tests or Mann–Whitney *U* tests were applied to compare the total cohort with patients who completed all three time points. The choice between parametric and non‐parametric tests was determined by assessing the data normality using the Shapiro–Wilk test.

### 2.6 Longitudinal Modeling

The longitudinal trajectories of chemotherapy‐induced changes were assessed using linear mixed‐effects models (LMEs; implemented via the *lme4* and *lmerTest* packages). LMEs were fitted using restricted maximum likelihood estimation to accommodate data missing at random. The modeling framework specifically evaluated temporal dynamics across three time points (bc1, bc2, bc3) in three domains: (i) MRI‐derived glymphatic function metrics (CP_ICV_, PVSVF‐WM, FW‐WM, DTI‐ALPS index); (ii) structural brain volume (ICV, WM/ICV, GM_cortex/ICV, GM_subcortex/ICV, CSF/ICV); and (iii) neuropsychological assessments (SAS, SDS, FACT‐Cog, DST, TMT‐A, VFT). Time points were modeled as fixed categorical effects, with adjustments for baseline age, years of education, mean arterial pressure (MAP), and body mass index (BMI). For the analysis of CP_ICV_, lateral ventricular volume was additionally included as a covariate to account for its potential influence on choroid plexus visualization and measurement. Bonferroni correction was applied to control for multiple comparisons within each domain. For those indicators that have longitudinal changes after multiple comparisons in the LMEs, pairwise comparisons between time points were performed post hoc using estimated marginal means (*emmeans* package, v1.10.5), with Bonferroni correction to account for multiple testing.

### 2.7 Correlation Analysis

Correlation analyses were performed for parameters that remained statistically significant after multiple comparison correction in the LMEs. Specifically, we calculated correlations among parameters at each individual time point, as well as correlations between the longitudinal changes (i.e., differences) of these parameters across time points. The Shapiro–Wilk test was used to assess the normality of continuous variables. Pearson correlation was applied to normally distributed data, while Spearman correlation was used for non‐normally distributed variables. To control for potential confounders, baseline age, education, MAP, and BMI were included as covariates.

### 2.8 Cross‐Lagged Panel Analysis

To examine temporal precedence among MRI‐derived glymphatic function metrics that showed significant longitudinal changes during neoadjuvant chemotherapy, cross‐lagged panel analysis (*lavaan* package, v0.6.19) was conducted on MRI‐derived glymphatic variables that survived multiple comparison correction in the LMEs. The model included both autoregressive paths and cross‐lagged paths, while adjusting for age, education, MAP, and BMI. Details are provided in Appendix S1.

## 3 Results

### 3.1 Study Participants

**FIGURE 2:**
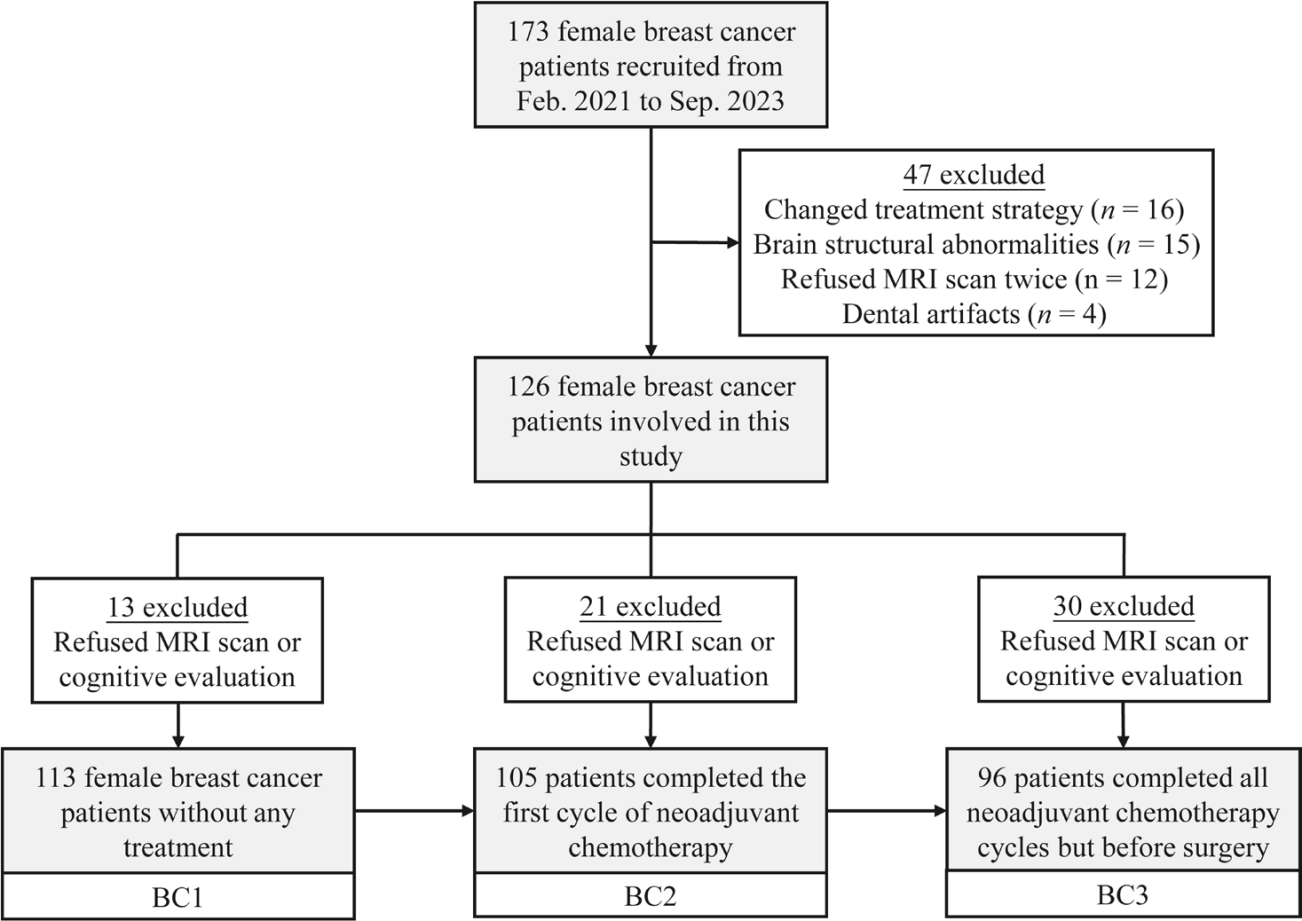
Study flowchart.

A study flowchart is presented in Figure 2. Among the 173 initially recruited breast cancer patients, 16 were excluded due to changes in treatment strategy, 15 due to brain metastases (*n* = 9) or severe white matter hyperintensities (*n* = 6), 12 due to missing longitudinal MRI scans, and 4 due to dental artifacts. Ultimately, a total of 126 patients were included in this study. Among them, 113 completed brain MRI and neuropsychological assessments at bc1, 105 at bc2 (29.8 ± 7.4 days from bc1), and 96 at bc3 (156.7 ± 25.4 days from bc1). Demographic and clinical characteristics of the included patients are summarized in Table 1. No significant clinicopathological differences were observed between the overall cohort and the subsets assessed at each of the three timepoints (*p* = 0.80–0.99).

**TABLE 1:** Clinical‐pathologic participant characteristics.

Characteristics	Total included patients (*n* = 126)	BC1 (*n* = 113)	bc2 (*n* = 105)	BC3 (*n* = 96)	*P*
Age (years)^a^	49.2 ± 9.3	48.9 ± 9.4	49.5 ± 9.6	48.9 ± 8.8	0.92
Education (years)^a^	8.8 ± 3.6	8.8 ± 3.7	8.8 ± 3.8	8.6 ± 3.5	0.99
MAP^a^	97.9 ± 11.1	97.9 ± 11.4	98.1 ± 10.9	97.4 ± 11.0	0.97
BMI^a^	24.5 ± 3.3	24.5 ± 3.4	24.2 ± 3.4	24.6 ± 3.3	0.80
Days after therapy^a^	—	—	29.8 ± 7.4	156.7 ± 25.4	
Cancer stage					
II	29 (23.0)	24 (21.2)	23 (21.9)	22 (22.9)	0.99
III	97 (77.0)	89 (78.8)	82 (78.1)	74 (77.1)	
Molecular subtype					
Luminal A	10 (7.9)	9 (8.0)	8 (7.6)	7 (7.3)	0.99
Luminal B	69 (54.8)	61 (54.0)	60 (57.1)	54 (56.3)	
*HER2*‐enriched	24 (19.0)	21 (18.6)	17 (16DTI‐.2)	21 (21.9)	
TNBC	23 (18.3)	22 (19.5)	20 (19.0)	14 (14.6)	
Therapy regimens					
Anthracycline‐based	2 (1.6)	—	2 (1.9)	2 (2.1)	0.99
Taxane‐based	49 (38.9)	—	38 (36.2)	37 (38.5)	
Anthracycline and taxane‐based	75 (59.5)	—	65 (61.9)	57 (59.4)	
Target therapy					
Yes	49 (38.9)	—	37 (35.2)	40 (41.7)	0.92
No	77 (61.1)	—	68 (64.8)	56 (58.3)	

*Note:* Unless otherwise indicated, data are numbers of participants; data in parentheses are percentages.

Abbreviations: BMI, body mass index; *HER2*, human epidermal growth factor receptor 2; MAP, mean arterial pressure; TNBC, triple‐negative breast cancer.

^a^ Data are means ± SDs for continuous variables.

Of the 126 patients, 63 completed assessments at all three time points, while the remainder completed assessments at two time points. The missing data were mainly attributable to the time and financial burden of brain imaging procedures and appeared to be largely random. No significant clinicopathological differences were found between the full cohort and those with complete data (*p* = 0.33–0.92; Table S1). Five participants had a history of diabetes, all of whom maintained well‐controlled blood glucose levels throughout the study period under regular treatment. All participants were non‐smokers, with 32.5% reporting household exposure to second‐hand smoke. At each time point, all patients received corticosteroids as part of routine supportive care.

### 3.2 Longitudinal Changes of Measured Parameters During Neoadjuvant Chemotherapy

**FIGURE 3:**
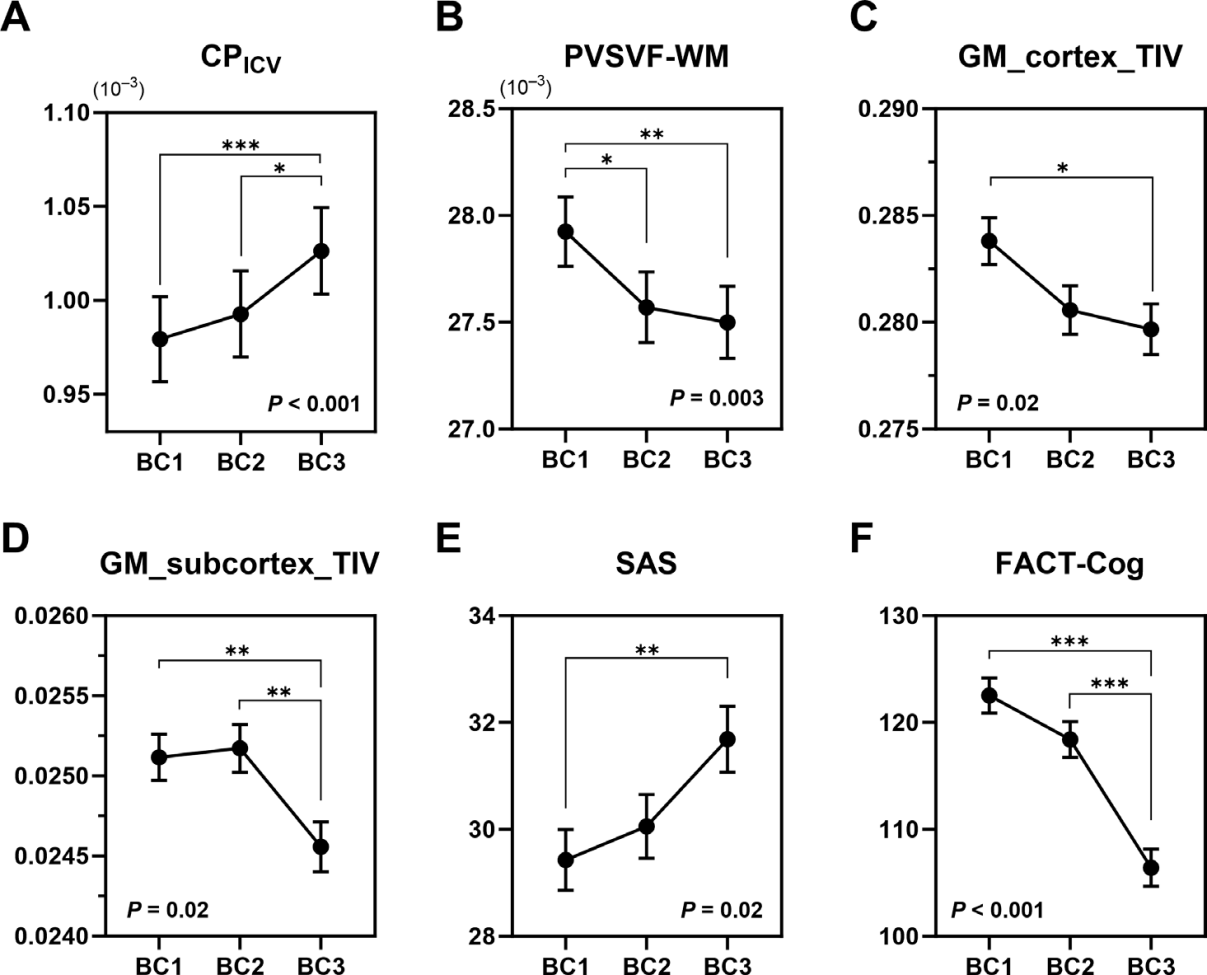
Longitudinal trajectories of variables with significant effects in linear mixed‐effects models after Bonferroni correction. Panels show estimated marginal means ± standard errors at three time points (bc1, bc2, bc3) across domains: (A) MRI‐derived glymphatic function metrics; (B) structural brain volumes; and (C) neuropsychological assessments. All *p*‐values shown in the figure reflect Bonferroni‐corrected results. *p*‐values for post hoc pairwise comparisons (Bonferroni corrected) are indicated as follows: *P* < 0.05 (*), *p* < 0.01 (**), *p* < 0.001 (***). bc1 = baseline (pre‐treatment), bc2 = after the first cycle of neoadjuvant chemotherapy, bc3 = after completion of neoadjuvant chemotherapy but prior to surgery, CP_ICV_ = ratio of choroid plexus volume to intracranial volume, PVSVF‐WM, volume fraction of perivascular space in white matter, WM/ICV = ratio of white matter volume to intracranial volume, GM_cortex/ICV = ratio of cortical gray matter volume to intracranial volume, GM_subcortex/ICV = ratio of subcortical gray matter volume to intracranial volume, CSF/ICV = ratio of white matter cerebrospinal fluid volume to intracranial volume, SAS = Self‐Rating Anxiety Scale, FCAT‐Cog = Functional Assessment of Cancer Therapy‐Cognitive Function.

For MRI‐derived glymphatic function metrics, CP_ICV_ increased (Bonferroni‐corrected *p* < 0.001, Figure 3A) and PVSVF‐WM decreased (Bonferroni‐corrected *p* = 0.003, Figure 3B) significantly following chemotherapy (Table 2). In contrast, FW‐WM and DTI‐ALPS showed no significant changes during chemotherapy.

**TABLE 2:** Longitudinal changes of measured parameters during neoadjuvant chemotherapy in linear mixed model.

Parameter	*b* (SE)	*p*	Bonferroni‐corrected *p*
MRI‐derived glymphatic function metrics			
CP_ICV_ (10^−3^)	0.023 (0.006)	**< 0.001**	**< 0.001**
PVSVF‐WM (10^−3^)	−0.219 (0.065)	**< 0.001**	**0.003**
FW‐WM (10^−3^)	0.736 (0.573)	0.20	0.80
DTI‐ALPS (10^−3^)	−7.588 (5.207)	0.15	0.59
Structural brain volumetry			
ICV (cm^3^)	0.551 (2.591)	0.92	1
WM/ICV (10^−3^)	−0.133 (0.761)	0.86	1
GM_cortex/ICV (10^−3^)	−2.113 (0.718)	**0.004**	**0.02**
GM_subcortex/ICV (10^−3^)	−0.267 (0.091)	**0.004**	**0.02**
CSF/ICV (10^−3^)	3.624 (1.578)	**0.02**	0.11
Neuropsychological assessments			
SAS	1.103 (0.358)	**0.002**	**0.02**
SDS	0.755 (0.445)	0.09	0.64
FACT‐Cog	−7.924 (1.335)	**< 0.001**	**< 0.001**
DST, forward	−0.066 (0.091)	0.47	1
DST, backward	−0.126 (0.064)	0.052	0.36
TMT‐A	−0.422 (1.606)	0.79	1
VFT	−0.056 (0.385)	0.88	1

*Note:* Bold = significant at *p* < 0.05.

Abbreviations: DTI–ALPS, Diffusion Tensor Imaging–analysis along the PVS space; CP_ICV_, ratio of choroid plexus volume to intracranial volume; CSF/ICV, ratio of cerebrospinal fluid volume to intracranial volume; DST, the Digit Span Test; FCAT‐Cog, Functional Assessment of Cancer Therapy‐Cognitive Function; FW‐WM, free water in white matter; GM_cortex/ICV, ratio of cortical gray matter volume to intracranial volume; GM_subcortex/ICV, ratio of subcortical gray matter volume to intracranial volume; ICV, intracranial volume; PVSVF‐WM, PVS volume fraction in white matter; SAS, Self‐Rating Anxiety Scale; SDS, Self‐Rating Depression Scale; TMT‐A, the Trail Making Test parts A; VFT, the Verbal Fluency Test; WM/ICV, ratio of white matter volume to intracranial volume.

Regarding structural brain volume, the GM_cortex/ICV and GM_subcortex/ICV ratios significantly decreased (both Bonferroni‐corrected *p* = 0.02, Figure 3C,D), while ICV, WM/ICV, and CSF/ICV showed no significant changes during chemotherapy (Table 2).

For neuropsychological assessments, SAS scores significantly increased (Bonferroni‐corrected *p* = 0.02, Figure 3E), while FACT‐Cog scores significantly decreased (Bonferroni‐corrected *p* < 0.001, Figure 3F) after chemotherapy (Table 2). No significant changes were observed in SDS, DST, TMT‐A, or VFT scores during chemotherapy.

Post hoc pairwise comparisons between time points are summarized in Table 3.

**TABLE 3:** Post hoc pairwise comparisons between time points.

Parameter	BC1	BC2	BC3	BC1‐BC2		BC2‐BC3		BC1‐BC3	
				*p*	Corrected *p*	*p*	Corrected *p*	*p*	Corrected *p*
MRI‐derived glymphatic function metrics									
CP_ICV_ (10^−3^)	0.979 (0.023)	0.993 (0.023)	1.026 (0.023)	0.22	0.65	**0.004**	**0.01**	**< 0.001**	**< 0.001**
PVSVF‐WM (10^−3^)	27.925 (0.163)	27.570 (0.166)	27.499 (0.169)	**0.005**	**0.01**	0.60	1	**0.001**	**0.004**
Structural brain volumetry									
GM_cortex/ICV	0.2838 (0.0011)	0.2806 (0.0011)	0.2797 (0.0012)	**0.02**	0.07	0.54	1	**0.005**	**0.01**
GM_subcortex/ICV	0.0251 (0.0001)	0.0252 (0.0001)	0.0246 (0.0002)	0.75	1	**0.001**	**0.003**	**0.002**	**0.007**
Neuropsychological assessments									
SAS	29.427 (0.571)	30.057 (0.598)	31.668 (0.614)	0.37	1	**0.03**	0.09	**0.002**	**0.006**
FACT‐Cog	122.517 (1.637)	118.407 (1.674)	106.407 (1.747)	0.053	0.16	**< 0.001**	**< 0.001**	**< 0.001**	**< 0.001**

*Note:* Data are marginal means (SEs). Bold = significant at *p* < 0.05.

Abbreviations: CP_ICV_, ratio of choroid plexus volume to intracranial volume; FCAT‐Cog, Functional Assessment of Cancer Therapy‐Cognitive Function; GM_cortex/ICV, ratio of cortex volume to intracranial volume; GM_subcortex/ICV, ratio of subcortex volume to intracranial volume; PVSVF‐WM, PVS volume fraction in white matter; SAS, Self‐Rating Anxiety Scale.

### 3.3 Correlations Among Parameters Following Neoadjuvant Chemotherapy

**FIGURE 4:**
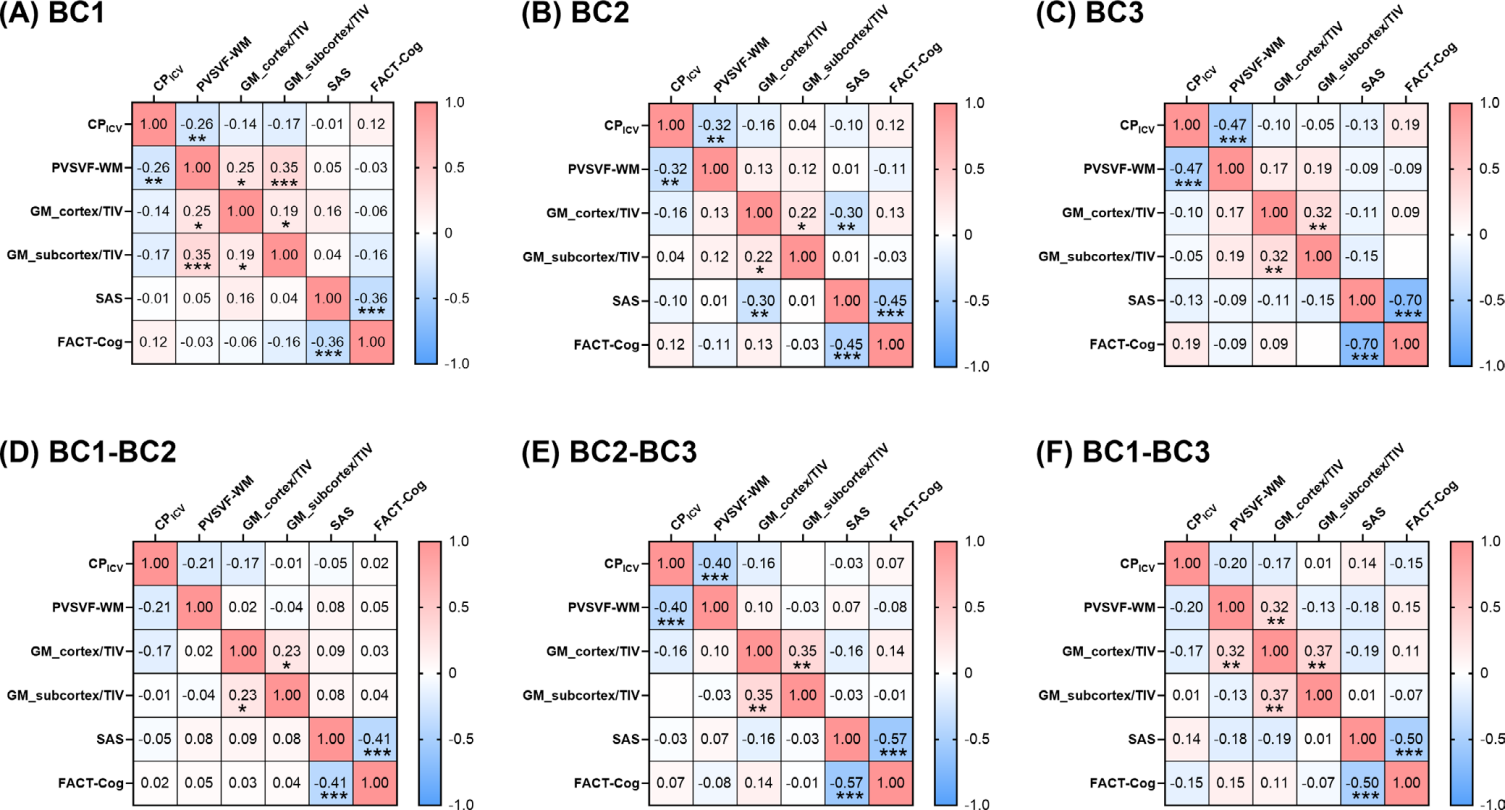
Correlation analysis matrices for variables with significant longitudinal changes. Panels show cross‐sectional correlations among these variables at each time point: (A) bc1, (B) bc2, and (C) bc3; and correlations of their longitudinal changes between time points: (D) bc1–bc2, (E) bc2–bc3, and (F) bc1–bc3. bc1 = baseline (pre‐treatment), bc2 = after the first cycle of neoadjuvant chemotherapy, bc3 = after completion of neoadjuvant chemotherapy but prior to surgery, CP_ICV_ = ratio of choroid plexus volume to intracranial volume, PVSVF‐WM, volume fraction of perivascular space in white matter, GM_cortex/ICV = ratio of cortical gray matter volume to intracranial volume, GM_subcortex/ICV = ratio of subcortical gray matter volume to intracranial volume, SAS = Self‐Rating Anxiety Scale, FCAT‐Cog = Functional Assessment of Cancer Therapy‐Cognitive Function. *p < 0.05, **p < 0.01, ***p < 0.001.

**FIGURE 5:**
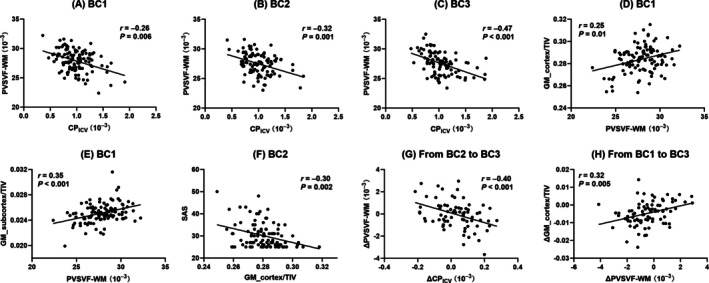
Scatter plots showing correlations among variables with significant longitudinal changes. (A–C) Correlations between CP_ICV_ and PVSVF‐WM at each time point; (D–E) Baseline correlations between PVSVF‐WM and structural brain volumes; (F) Correlation between SAS scores and GM_cortex/ICV at bc2; (G) Correlation between longitudinal changes in CP_ICV_ and PVSVF‐WM from bc2 to bc3; (H) Correlation between longitudinal changes in PVSVF‐WM and GM_cortex/ICV from bc1 to bc3. bc1 = baseline (pre‐treatment), bc2 = after the first cycle of neoadjuvant chemotherapy, bc3 = after completion of neoadjuvant chemotherapy but prior to surgery, CP_ICV_ = ratio of choroid plexus volume to intracranial volume, PVSVF‐WM, volume fraction of perivascular space in white matter, GM_cortex/ICV = ratio of cortical gray matter volume to intracranial volume, GM_subcortex/ICV = ratio of subcortical gray matter volume to intracranial volume, SAS = Self‐Rating Anxiety Scale. *p < 0.05, **p < 0.01, ***p < 0.001.

The correlation matrices for each time point are presented in Figure 4A–C. At each time point, a number of significant correlations were observed within each domain, including (i) MRI‐derived glymphatic function metrics (CP_ICV_ and PVSVF‐WM), (ii) structural brain volume measures (GM_cortex/ICV and GM_subcortex/ICV), and (iii) neuropsychological assessments (SAS and FACT‐Cog). Notably, the negative correlation between CP_ICV_ and PVSVF‐WM progressively strengthened over the course of chemotherapy, with correlation coefficients of *r* = −0.26 (*p* = 0.006) at bc1, *r* = −0.32 (*p* = 0.001) at bc2, and *r* = −0.47 (*p* < 0.001) at bc3, respectively (Figure 5A–C). Additionally, PVSVF‐WM showed significant associations with GM_cortex/ICV (*r* = 0.25, *p* = 0.01, Figure 5D) and GM_subcortex/ICV (*r* = −0.35, *p* < 0.001, Figure 5E) at bc1. GM_cortex/ICV showed significant associations with SAS (*r* = −0.30, *p* = 0.002, Figure 5F) at bc2. No significant correlations were found between other cross‐domain parameters.

Moreover, correlation analyses based on longitudinal changes revealed that increases in CP_ICV_ from bc2 to bc3 were negatively correlated with decreases in PVSVF‐WM (*r* = −0.40, *p* < 0.001, Figure 5G). From bc1 to bc3, reductions in PVSVF‐WM were positively associated with decreases in GM_cortex/ICV (*r* = 0.32, *p* = 0.005, Figure 5H). In addition, GM_cortex/ICV and GM_subcortex/ICV, as well as SAS and FACT‐Cog scores, exhibited significant correlations in all three longitudinal intervals (Figure 4D–F).

### 3.4 Temporal Precedence Between CPICV and PVSVF‐WM During Neoadjuvant Chemotherapy

**FIGURE 6:**
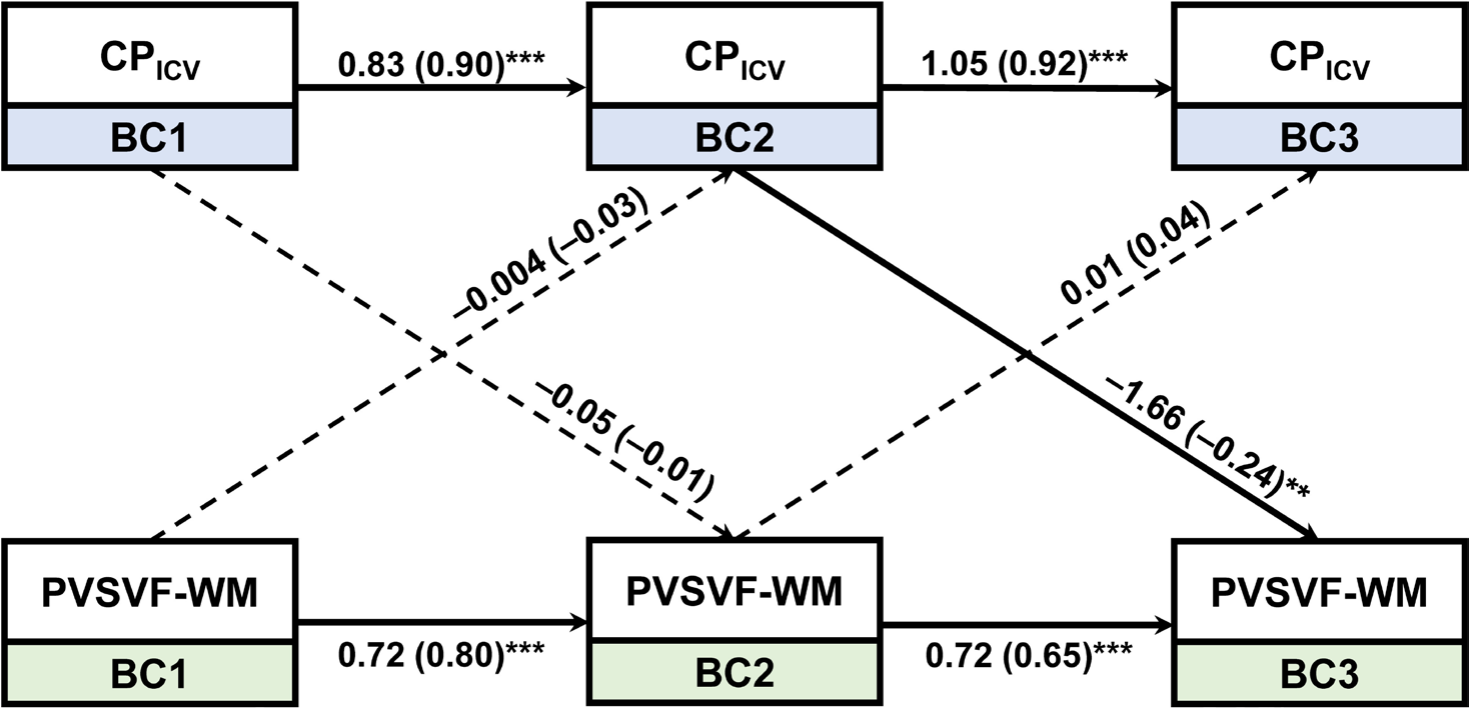
Cross‐lagged panel model illustrating the temporal associations between variables across three time points. Path coefficients are shown as unstandardized estimates, with standardized values provided in parentheses. Solid arrows represent statistically significant paths (*p* < 0.05), while dashed arrows indicate nonsignificant paths. bc1 = baseline (pre‐treatment), bc2 = after the first cycle of neoadjuvant chemotherapy, bc3 = after completion of neoadjuvant chemotherapy but prior to surgery, CP_ICV_ = ratio of choroid plexus volume to intracranial volume, PVSVF‐WM, volume fraction of perivascular space in white matter. *p < 0.05, **p < 0.01, ***p < 0.001.

The autoregressive effects were statistically significant for both variables across adjacent time points (all *p* < 0.001). Notably, a significant cross‐lagged effect was observed from CP_ICV_ at bc2 to PVSVF‐WM at bc3 (*β* = −1.66, *p* = 0.007) (Figure 6).

## 4 Discussion

This longitudinal study systematically evaluated glymphatic function, brain structural volume, and neuropsychological performance during neoadjuvant chemotherapy in breast cancer patients. Among MRI‐derived glymphatic metrics, CP_ICV_ significantly increased, while PVSVF‐WM significantly decreased over the treatment course. Structural MRI revealed reductions in GM_cortex/ICV and GM_subcortex/ICV. Psychologically, patients exhibited elevated SAS and decreased FACT‐Cog scores following chemotherapy. Correlation analyses based on longitudinal changes showed that increases in CPICV were associated with decreases in PVSVF‐WM, and reductions in PVSVF‐WM were associated with decreases in GM_cortex/ICV. Cross‐lagged panel analysis further indicated a significant temporal relationship from CP_ICV_ at bc2 to PVSVF‐WM at bc3.

Among the MRI‐derived glymphatic metrics, CP_ICV_ increased while PVSVF‐WM decreased significantly over the course of neoadjuvant chemotherapy. Chemotherapy has been shown to induce oxidative stress–mediated injury to the choroid plexus (Jang et al. 2022). Although changes in CP volume following chemotherapy have not been well studied, prior work has linked choroid plexus enlargement with reduced glymphatic function (Li et al. 2023). The underlying mechanisms may involve increased BCSFB permeability and neuroinflammation, as observed in conditions such as multiple sclerosis and Alzheimer's disease (Bouhrara et al. 2024; Fleischer et al. 2021). As chemotherapy is also known to induce neuroinflammatory responses, it may contribute to choroid plexus enlargement through similar mechanisms (Briones and Woods 2014). Such choroid plexus enlargement in disease conditions has been reported to be potentially accompanied by reduced CSF production (Damkier et al. 2013). Although reductions in PVS volume fraction found in our study are rarely observed in neurodegenerative diseases (Kamagata et al. 2022; Lin et al. 2024), they have been reported following chemotherapy in a recent study, which documented significant decreases in pediatric medulloblastoma patients (Song et al. 2025). This observation is consistent with our findings and supports the possibility that chemotherapy may contribute to PVS shrinkage. In our study, the concurrent increase in CP_ICV_ and decrease in PVSVF‐WM may reflect this mechanism: structural enlargement of the choroid plexus, potentially driven by chemotherapy‐induced inflammatory or toxic effects, could impair CSF secretory function. Reduced CSF production may lead to diminished CSF influx into the perivascular spaces of the white matter, manifesting as a lower PVSVF‐WM. The observed negative correlation between CP_ICV_ and PVSVF‐WM supports this interpretation and may represent a feature of chemotherapy‐related brain alterations.

The cross‐lagged panel analysis provided temporal evidence that changes in CP_ICV_ preceded subsequent alterations in PVSVF‐WM. This temporal ordering is consistent with the mechanism hypothesized in the preceding paragraph, suggesting that choroid plexus alterations during chemotherapy may be followed by downstream changes in perivascular spaces (Xu, Wang, et al. 2024; Lun et al. 2015). Such a directional relationship provides preliminary evidence for sequential disruptions within the glymphatic pathway during neoadjuvant treatment. However, while temporal precedence was observed, these results should not be interpreted as evidence of a causal relationship. Future studies with larger samples and multimodal imaging approaches are needed to confirm this temporal pattern and to clarify the potential mechanisms linking choroid plexus changes with perivascular space alterations.

In addition, no significant changes were observed in the FW‐WM (which reflects CSF–ISF exchange) or the DTI‐ALPS index (which reflects ISF clearance from the PVS) during neoadjuvant chemotherapy. This suggests that despite the impact on upstream CSF production and inflow into the PVS, the downstream exchange and clearance processes remained functionally preserved over the course of chemotherapy. Notably, previous studies have similarly reported stable DTI‐ALPS values following neoadjuvant chemotherapy (Gao et al. 2024). When considered together with the observed increase in CP_ICV_ and decrease in PVSVF‐WM, this pattern suggests that chemotherapy may preferentially affect the upstream CSF production and inflow phases of the glymphatic pathway, while having less impact on the downstream efflux phase from the PVS during the early chemotherapy period. It is also possible that the FW‐WM and DTI‐ALPS index have limited sensitivity to detect early or subtle changes under chemotherapy.

Significant changes in structural brain volumes were observed, with both GM_cortex/ICV and GM_subcortex/ICV ratios showing reductions during chemotherapy, while ICV, WM/ICV, and CSF/ICV remained stable. These findings are consistent with previous studies reporting gray matter volume loss in breast cancer survivors following chemotherapy (Nikolaeva et al. 2025; de Ruiter et al. 2023). Importantly, gray matter volumes were significantly correlated with PVSVF‐WM, both at baseline and in their longitudinal changes. It suggests that disruptions in glymphatic function may be closely linked to changes in brain structure during neoadjuvant chemotherapy.

This study identified significant changes in SAS and FACT‐Cog scores during neoadjuvant chemotherapy, with higher SAS scores indicating increased anxiety and lower FACT‐Cog scores reflecting greater self‐reported cognitive decline. Notably, both are self‐reported measures, whereas objective neuropsychological tests (DST, TMT‐A, and VFT) did not show significant changes over the treatment course. Consistent with this interpretation, self‐reported cognitive decline may reflect psychological distress rather than measurable cognitive dysfunction (Hutchinson et al. 2012), as supported by the strong negative correlation between SAS and FACT‐Cog scores observed in this study. It is also possible that the neuropsychological battery used here was not sufficiently sensitive to detect subtle early changes in cognitive function, and practice effects may have further masked minor changes, particularly in objective cognitive measures (Schagen et al. 2012).

Regarding the relationship between glymphatic function and cognition, we did not find significant correlations between changes in MRI‐derived glymphatic metrics and cognitive scores after chemotherapy. This lack of association may reflect a temporal dissociation in which glymphatic alterations occur earlier than detectable changes in objective cognitive tests. Moreover, changes in subjective cognitive function may be influenced by multiple factors, including emotional state and fatigue, which could obscure any direct relationship with glymphatic measures (Kim and Abraham 2021). In addition to these findings, we observed that higher SAS scores were significantly associated with lower GM_cortex/ICV at bc2, suggesting that anxiety during chemotherapy may also be linked to structural brain changes (Chen et al. 2020).

This study has several notable strengths. First, a relatively large sample size and a longitudinal design that allowed for the dynamic assessment of chemotherapy‐related changes. Second, a comprehensive evaluation of glymphatic system alterations following neoadjuvant chemotherapy (including CP_ICV_, PVSVF‐WM, FW‐WM, and DTI‐ALPS) integrated with structural brain volume measures and neuropsychological assessments. Third, the identification of longitudinal temporal patterns and trajectories of glymphatic changes post‐chemotherapy.

Our study has several limitations. First, although it provides a comprehensive observational characterization of glymphatic system alterations following chemotherapy, it does not explore the underlying mechanisms by which chemotherapy may impair glymphatic function. Future studies are warranted to investigate these pathophysiological pathways. Second, the follow‐up duration was relatively short, and the absence of significant changes in some glymphatic indices (e.g., FW‐WM and DTI‐ALPS) should be interpreted with caution. Long‐term longitudinal studies are needed to determine whether delayed effects may emerge over time. Third, susceptibility‐weighted imaging (SWI) sequences were not acquired in this study, which may have limited the precision of medullary vein visualization for DTI‐ALPS ROI placement. Fourth, sleep patterns were not assessed, and given that patients may experience anxiety after chemotherapy, potential sleep disturbances could have influenced glymphatic function measurements. Fifth, blood lipid levels were not measured in this study, which may have influenced the interpretation of glymphatic function, as dyslipidemia can affect cerebrovascular integrity and interstitial fluid dynamics. Finally, the lack of a healthy control group limits the ability to distinguish treatment‐related changes from normative fluctuations. In addition, due to ethical constraints, we were unable to include a control group of breast cancer patients who did not receive chemotherapy at all assessed time points. Although the use of a mixed‐effects model enabled robust analysis across multiple time points in this exploratory longitudinal design, the lack of control groups limits the generalizability of our findings.

## 5 Conclusion

During neoadjuvant chemotherapy, breast cancer patients exhibited increased choroid plexus volume and decreased perivascular space volume fraction, which were associated with chemotherapy‐induced gray matter atrophy. Early alterations in choroid plexus volume may precede subsequent changes in the perivascular space. These findings suggest that the glymphatic system may play a role in the pathophysiology of chemotherapy‐related brain changes and offer a potential target for early intervention.

## Conflicts of Interest

The authors declare no conflicts of interest.

## Supporting information


**Data S1:** hbm70334‐sup‐0001‐AppendixS1‐TableS1.docx.

## Data Availability

The data in this manuscript are a part of a longitudinal study that will generate more than one manuscript. The data will be available upon completion of these manuscripts. Original data can be made available upon reasonable request.
