# Supplementary material for: Glymphatic, Structural, and Cognitive Changes During Breast Cancer Chemotherapy: A Longitudinal MRI Study
Source: Hum Brain Mapp. 2025 Aug 29;46(13):e70334. doi: 10.1002/hbm.70334 (PMC12397493; doi:10.1002/hbm.70334)
Supplement: Supplementary file 1 — Data S1: hbm70334‐sup‐0001‐AppendixS1‐TableS1.docx. [file HBM-46-e70334-s001.docx]

| **Supplementary Materials** |
| --- |

**Appendix S1**

**Cross-Lagged Panel Model:**

The cross-lagged panel model (CLPM) was constructed to explore potential directional influences among MRI-derived glymphatic function metrics that demonstrated significant longitudinal changes and survived multiple comparison correction in linear mixed-effects models. All analyses were performed using the *lavaan* package (v0.6.19) in R.

The model incorporated three time points corresponding to baseline (pre-treatment, BC1), after the first cycle of neoadjuvant chemotherapy (BC2), and after completion of neoadjuvant chemotherapy but prior to surgery (BC3). The included glymphatic variables were CP_ICV_ (a ratio of the choroid plexus volume relative to the total intracranial volume) and PVSVF-WM (perivenous space volume fraction in white matter), both of which survived multiple comparison correction in linear mixed-effects models.

To enhance the robustness of the cross-lagged modeling and ensure the stability of parameter estimation, this analysis included only participants with complete data across all three time points. Clinical and pathologic characteristics of this subgroup were comparable to those of the overall sample, with no significant differences observed (all *P*-values = 0.33–0.96; Table S1).

The model incorporated both autoregressive and cross-lagged paths. Autoregressive paths were defined for each variable across adjacent time points, reflecting temporal stability:

CP_ICV__BC2 ~ CP_ICV__BC1

CP_ICV__BC3 ~ CP_ICV__BC2

PVSVF_BC2 ~ PVSVF_BC1

PVSVF_BC3 ~ PVSVF_BC2

Cross-lagged paths were modeled to test potential directional effects between variables:

PVSVF_BC2 ~ CP_ICV__BC1

CP_ICV__BC2 ~ PVSVF_BC1

PVSVF_BC3 ~ CP_ICV__BC2

CP_ICV__BC3 ~ PVSVF_BC2

This full cross-lagged structure allows for the estimation of potential unidirectional or reciprocal influences between CPICV and PVSVF-WM over time.

To account for shared variance between the two glymphatic variables measured at the same time point, residual covariances were estimated between CP_ICV_ and PVSVF-WM at each session (BC1, BC2, and BC3). Furthermore, baseline values of all glymphatic metrics were regressed on exogenous covariates including age, education (years), mean arterial pressure (MAP), and body mass index (BMI), allowing control for demographic and clinical differences at baseline.

All parameters were estimated using full information maximum likelihood (FIML). Model fit was assessed using standard indices including the Comparative Fit Index (CFI), Tucker-Lewis Index (TLI), Root Mean Square Error of Approximation (RMSEA), and Standardized Root Mean Square Residual (SRMR).

Path coefficients for both autoregressive and cross-lagged effects were extracted and interpreted to identify the dominant temporal directions of influence among glymphatic function metrics over the course of neoadjuvant chemotherapy.

**Table S1**

| **Table 1: Clinical-Pathologic Participant Characteristics** | | | |
| --- | --- | --- | --- |
| Characteristics | Total included patients (*n* = 126) | Patients with complete observations (*n* = 63) | *P* Value |
| Age (years)* | 49.2 ± 9.3 | 48.4 ± 9.4 | 0.72 |
| Education (years)* | 8.8 ± 3.6 | 8.5 ± 3.9 | 0.55 |
| MAP | 97.9 ± 11.1 | 98.4 ± 11.8 | 0.74 |
| BMI | 24.5 ± 3.3 | 24.0 ± 3.3 | 0.33 |
| Cancer stage |  |  |  |
| Ⅱ | 29 (23.0) | 15 (23.8) | 0.90 |
| Ⅲ | 97 (77.0) | 48 (76.2) |  |
| Molecular subtype |  |  |  |
| Luminal A | 10 (7.9) | 5 (7.9) | 0.96 |
| Luminal B | 69 (54.8) | 37 (58.7) |  |
| *HER2*-enriched | 24 (19.0) | 11 (17.5) |  |
| TNBC | 23 (18.3) | 10 (15.9) |  |
| Therapy regimens |  |  |  |
| Anthracycline-based | 2 (1.6) | 2 (3.2) | 0.62 |
| Taxane-based | 48 (38.1) | 21 (33.3) |  |
| Anthracycline and taxane-based | 75 (59.5) | 40 (63.5) |  |
| Target therapy |  |  |  |
| Yes | 49 (38.9) | 25 (39.7) | 0.92 |
| No | 77 (61.1) | 38 (60.3) |  |
| Note.—Unless otherwise indicated, data are numbers of participants; data in parentheses are percentages. *HER2* = human epidermal growth factor receptor 2, TNBC = triple-negative breast cancer. * Data are means ± SDs for continuous variables. | | | |
